# Integrated analysis of a competing endogenous RNA network reveals an 11-lncRNA prognostic signature in ovarian cancer

**DOI:** 10.18632/aging.104116

**Published:** 2020-11-20

**Authors:** Yongjian Zhang, Hu Zhou, Meiyin Zhang, Linan Xing, Chang Yang, Bairong Xia, Ge Lou

**Affiliations:** 1 Department of Gynecology Oncology, Harbin Medical University Cancer Hospital, Harbin 150081, Heilongjiang Province, China; 2 Department of Gynecology Oncology, The First Affiliated Hospital of USTC, Division of Life Science and Medicine, University of Science and Technology of China, Hefei 230001, Anhui Province, China

**Keywords:** ovarian cancer, long noncoding RNA, ceRNA network, prognostic factor

## Abstract

Long noncoding RNA (lncRNA) can function as a competing endogenous RNA (ceRNA) involved in tumor initiation and progression. However, the prognostic roles of lncRNAs in the integrated analysis of the ceRNA network in ovarian cancer (OVC) are still lacking. This study aimed to identify lncRNAs associated with the prognosis of OVC. Differential expression analysis and WGCNA were used to screen OVC-specific RNAs. A lncRNA-miRNA-mRNA regulatory network consisting of 201 lncRNAs, 85 miRNA and 146 mRNAs was constructed, and functional enrichment and protein-protein network analyses were performed. Then, the OVC-specific RNAs were submitted to Cox regression analysis. Twelve differentially expressed lncRNAs and mRNAs were identified as significantly associated with OS of OVC patients. Meanwhile, 11 lncRNAs (including *C4A-AS1*, *LINC02408*, *LINC00488*) were established as prognostic risk formulas. The low-risk group had better OS and DFS than the high-risk group (*P* <0.01). Univariate and multivariate Cox regression analyses revealed the 11-lncRNA risk score as an independent prognostic factor. A prognostic nomogram was developed based on independent prognostic factors. Our data provide evidence that the 11-lncRNA signature could serve as an independent prognostic indicator. This study also suggests that these 11 lncRNAs potentially participate in the progression of OVC.

## INTRODUCTION

Ovarian cancer (OVC) is one of the most common gynecologic cancers worldwide, with increasing morbidity and mortality [1]. There are approximately 239,000 new diagnosed cases, accounting for 3.6% of new cases by population, and 152,000 deaths, accounting for 4.3% of cancer-related deaths in the population each year [1]. Although OVC patients can be managed by a series of therapeutic methods, including surgical resection, radiation therapy, chemotherapy and molecular-targeted therapy, the overall 5-year survival of OVC remains poor [2]. Moreover, owing to the lack of effective screening tests and early specific warning signs, most OVC patients are diagnosed at advanced stages [2]. Therefore, there is an urgent need to deeply understand the molecular mechanisms underlying the initiation and progression of OVC to improve early diagnosis, predict prognosis and develop effective therapeutics.

Salmena et al. first proposed the competing endogenous RNA (ceRNA) hypothesis in 2011 [3]. The central concept of the ceRNA hypothesis is that coding messenger RNAs (mRNAs) and noncoding RNAs (ncRNAs) share the same microRNA (miRNA) response elements (MREs) with miRNAs. ncRNAs act as natural miRNA "sponges" and inhibit the potential function of miRNAs on mRNAs through recognition and combining the MREs on miRNAs, which release miRNAs from target mRNAs and promote the expression of mRNAs [3, 4]. This novel molecular mechanism between ncRNAs and mRNAs has been proven to be involved in the initiation, progression, invasion and metastasis of cancers [5–7].

Long noncoding RNAs (lncRNAs), a subtype of ncRNAs, have attracted increasing attention in recent years. Accumulating evidence has demonstrated that lncRNAs contribute to the regulation of gene expression at the transcriptional and post-transcriptional levels and chromatin modifications [8–10]. Although the complete mechanisms of lncRNAs remain unclear, growing evidence supports that lncRNAs can function as ceRNAs that attract miRNAs to indirectly regulate the expression of target mRNA, influencing tumorigenesis and tumor progression [11–14]. Previous studies have shown that abnormal expression of lncRNAs can affect the biological process of OVC through the ceRNA approach [15–23]. However, most of these studies focused on a single lncRNA-miRNA-mRNA axis and were verified in limited sample sizes [15, 17–23]. Currently, there is still a lack of comprehensive analyses of the lncRNA-miRNA-mRNA regulatory network in OVC with large-scale sample sizes.

Here, we obtained RNA-Seq data and compared the differential expression profiles between 371 OVC samples obtained from The Cancer Genome Atlas (TCGA) database and 88 normal ovarian tissues obtained from Genotype-Tissue Expression (GTEx) database. The mRNAs and lncRNAs were also applied to weighted correlation network analysis (WGCNA) to enrich modules that were most related to OVC. Subsequently, the lncRNA-miRNA-mRNA regulatory network for OVC was constructed through an integrated analysis. Next, we performed an overall survival analysis of lncRNAs and mRNAs in the ceRNA network to identify OVC-related prognostic biomarkers, and a survival model with 11 target lncRNAs was established. Finally, a prediction nomogram based on the clinical features and 11 target lncRNA survival model was constructed to predict OVC prognosis.

## RESULTS

### Identification of significantly differentially expressed lncRNAs and mRNAs

**Figure 1:**
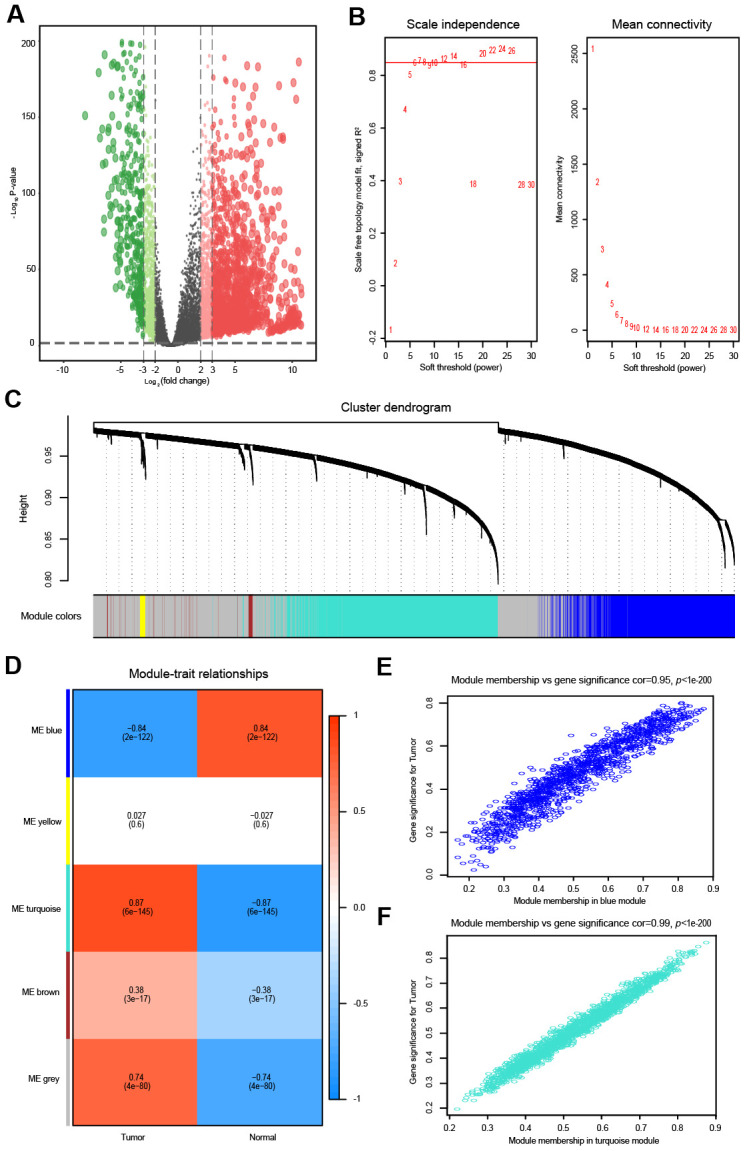
**Differential expression analysis and weighted correlation network analysis for the lncRNAs.** The volcano plots of differentially expressed lncRNAs. The green dots indicate significantly downregulated genes, the red dots indicate significantly upregulated genes, while the black dots indicate genes with no significant difference. (**A**) Identification of the soft threshold according to the standard of the scale-free network. The red line represents the threshold line of 0.85. (**B**) Clustering dendrogram of lncRNAs with dissimilarity based on the topological overlap together with assigned module colors. (**C**) Relationships between lncRNA modules and clinical traits. The correlation coefficient (upper number) and the corresponding P-value (lower number) in each cell resulted in the correlation between the lncRNA module and the clinical trait. (**D**) The scatterplot of gene significance vs. module membership in the lncRNA-based blue module. (**E**) The scatterplot of gene significance vs. module membership in the lncRNA-based turquoise module. (**F**) The scatterplot of gene significance vs. module membership in the lncRNA-based blue module.

**Figure 2:**
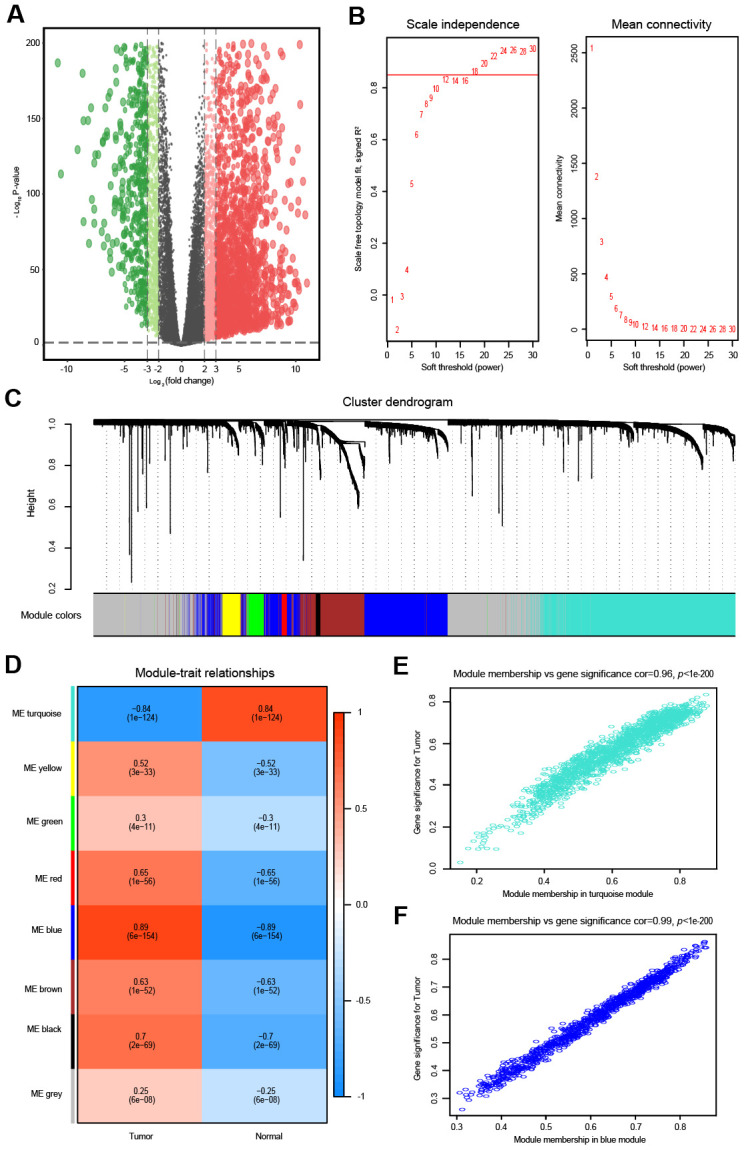
**Differential expression analysis and weighted correlation network analysis for the mRNAs.** (**A**) The volcano plots of differentially expressed mRNAs. The green dots indicate significantly downregulated genes, the red dots indicate significantly upregulated genes, while the black dots indicate genes with no significant difference. (**B**) Identification of the soft threshold according to the standard of the scale-free network. The red line represents the threshold line of 0.85. (**C**) Clustering dendrogram of mRNAs with dissimilarity based on the topological overlap together with assigned module colors. (**D**) Relationships between mRNA modules and clinical traits. The correlation coefficient (upper number) and corresponding P-value (lower number) in each cell resulted in the correlation between the mRNA module and the clinical trait. (**E**) The scatterplot of gene significance vs. module membership in the mRNA-based turquoise module. (**F**) The scatterplot of gene significance vs. module membership in the mRNA-based blue module.

According to the annotation information of Ensembl and miRbase databases, 14,148 lncRNAs, 19,645 mRNAs and 2,588 miRNAs were annotated. After removing the low-abundance genes, 8,860 lncRNAs, 18,438 mRNAs and 1,104 miRNAs were retained. Based on the given threshold, 3,279 differentially expressed lncRNAs (DElncRNAs), of which 2,210 DElncRNAs were upregulated and 1,069 DElncRNAs downregulated, and 4,789 differentially expressed mRNAs (DEmRNAs), of which 3,145 DEmRNAs were upregulated and 1,644 DEmRNAs downregulated, were identified between OVC tissues and normal tissues (Figure 1A and 2A).

### Weighted correlation network analysis

The top 5000 most variant lncRNAs, measured by the median absolute deviation (MAD), were selected for the WGCNA. To obtain a higher average connectivity degree in the lncRNA group, the soft threshold power was set to 7, which was the lowest threshold to make the scale-free R^2^ reach 0.85 (Figure 1B). Then, the cluster dendrogram was clustered based on the selected threshold, and 5 color modules were identified in the lncRNA group (Figure 1C). The lncRNAs that could not be included in any module were put into the gray module, which was removed in the subsequent analysis. The other four modules were represented in blue, yellow, turquoise and brown (Figure 1C). The relationship between modules and traits (OVC tissues and normal tissues) is shown in Figure 1D. Among these four modules, the turquoise module (R = 0.87, *P* < 0.01) and blue module (R = -0.84, *P* < 0.01) were significantly correlated with OVC (Figure 1D). Intra-modular analysis found that lncRNAs in the blue (R = 0.95, *P* < 0.01) and turquoise modules (R = 0.99, *P* < 0.01) were also highly correlated with OVC (Figure 1E, 1F).

Similarly, the top 5000 most variant mRNAs were selected for the WGCNA. As shown in Figure 2B, the soft threshold power was set to 18 to ensure a scale-free network, and we obtained eight modules for the next analysis (Figure 2C). Then, mRNAs in the eight color modules were continuously used to analyze the relationship between modules and traits (OVC tissues and normal tissues) (Figure 2D). The blue module (R = 0.89, *P* < 0.01) was positively associated with OVC, while the turquoise module (R = -0.84, *P* < 0.01) was negatively correlated with OVC (Figure 2D). Intra-modular analysis showed that mRNAs in both the turquoise module (R = 0.96, *P* < 0.01) and blue module (R = 0.99, *P* < 0.01) were highly related with OVC (Figure 2E, 2F).

### Functional enrichment analysis for the overlapped mRNAs

**Figure 3:**
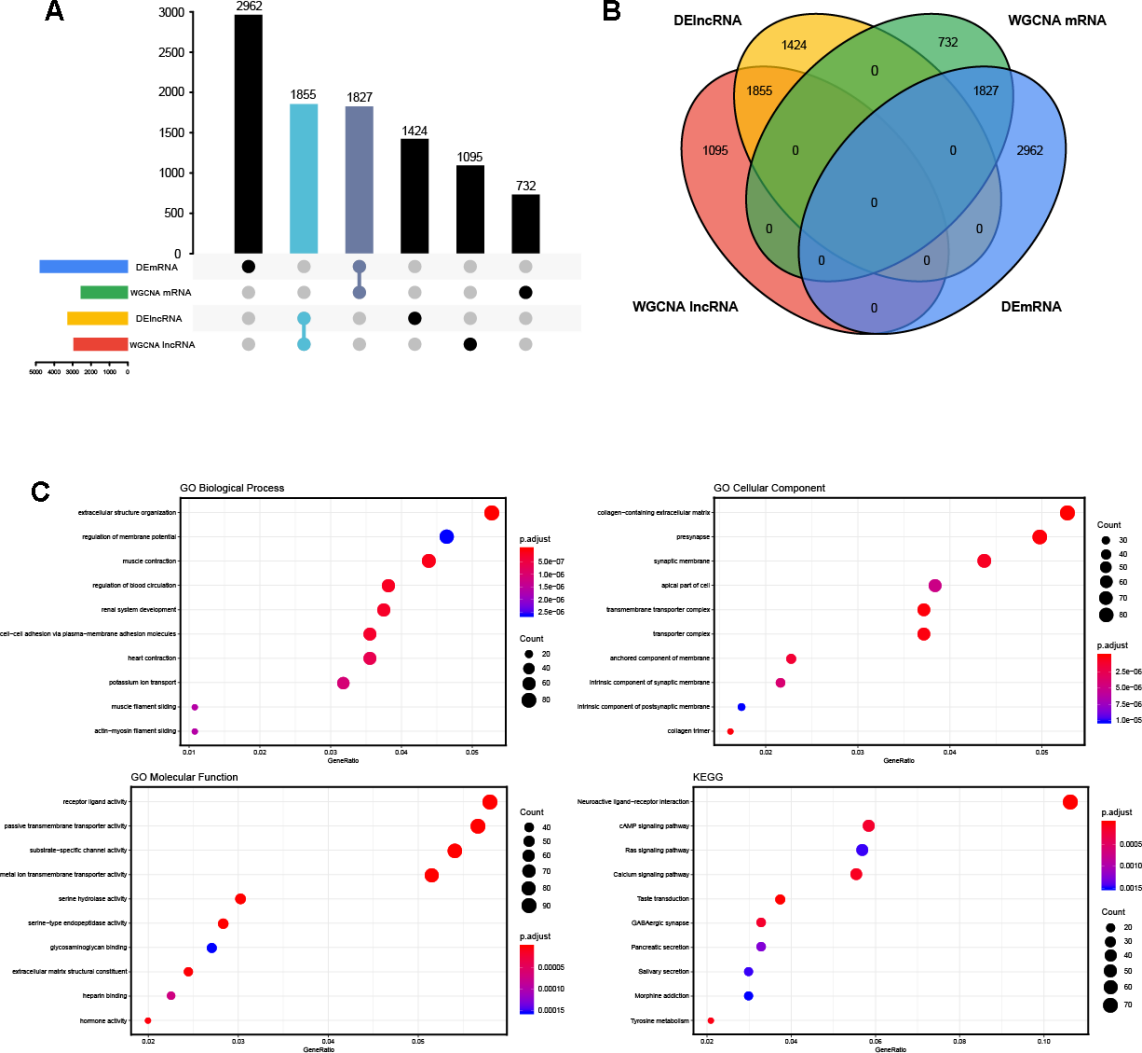
**The overlapped genes and enrichment analysis.** (**A, B**) The Venn diagram and the UpSet plots of overlapped genes between differential expression analysis and weighted correlation network analysis. (**C**) The top 10 significantly enriched Gene Ontology (GO) biological process (BP) terms, cellular component (CC) terms, molecular function (MF) terms and the Kyoto Encyclopedia of Genes and Genomes (KEGG) pathways.

The blue and turquoise modules, which were closely related to OVC, were selected and intersected with the above 4,789 DEmRNAs. In total, 1,827 mRNAs were obtained (Figure 3A, 3B). With a threshold of FDR < 0.01, GO enrichment analysis determined that 87 BP terms, 29 CC terms and 23 MF terms were significantly enriched in the overlapped mRNAs. KEGG pathway analysis revealed that 17 statistically significant signaling pathways were concentrated on the overlapped mRNAs. The top 10 terms of functional enrichment analysis are shown in Figure 3C.

### Construction of the lncRNA-miRNA-mRNA regulatory network

**Figure 4:**
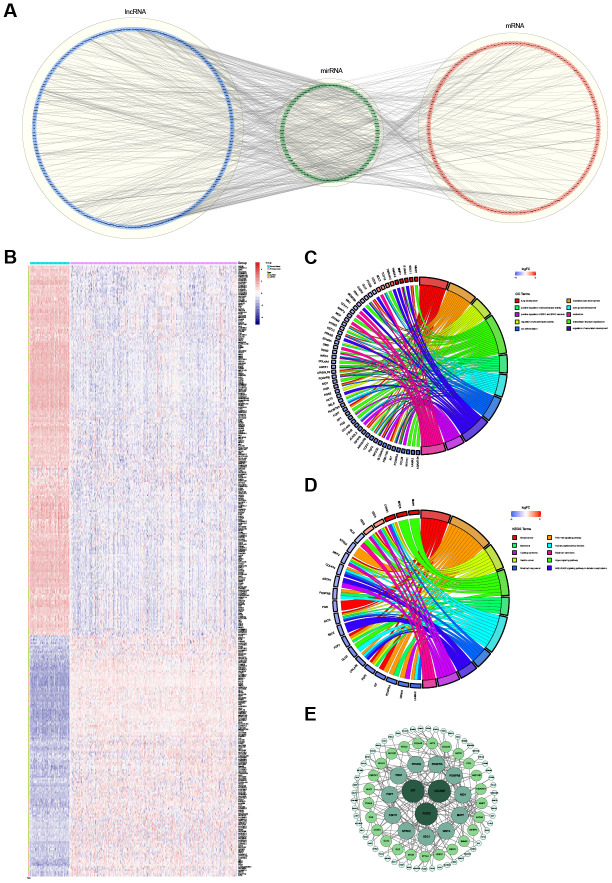
**The ceRNA regulatory network and functional analysis.** (**A**) The ceRNA network of lncRNA-miRNA-mRNA. The lncRNAs, miRNAs and mRNAs are indicated as blue, green and red, respectively. (**B**) The heatmap of the expression of 347 selected RNAs (201 lncRNAs and 146 mRNAs) in the ceRNA regulatory network. (**C**) The top 10 significantly enriched Gene Ontology (GO) biological process (BP) terms of mRNAs involved in the ceRNA regulatory network. (**D**) The top 10 significantly enriched Kyoto Encyclopedia of Genes and Genomes (KEGG) pathways of mRNAs involved in the ceRNA regulatory network. (**E**) The protein-protein interaction network. The greater the degree of the node, the bigger the node.

Overall, 1,885 lncRNAs, 1,827 mRNAs and 1,104 miRNAs were used to build the ceRNA regulatory network. According to the hypothesis of ceRNA, the candidate ceRNA triplets should meet the following criteria: (1) the lncRNA-mRNA interaction shows significantly positive correlations; (2) the miRNA-mRNA interaction and lncRNA-miRNA interaction show significantly negative correlations; (3) the lncRNA-miRNA-mRNA triplets show significant results in hypergeometric testing. Finally, the lncRNA-miRNA-mRNA ceRNA regulatory network, including 201 lncRNAs, 85 miRNAs and 146 mRNAs, was established based on the interactions of 487 lncRNA-miRNA pairs and 248 miRNA-mRNA pairs (Figure 4A). The expression of these 201 lncRNAs and 146 mRNAs between 371 OVC tissues and 88 normal tissues is shown in Figure 4B.

### Functional enrichment and PPI analyses of the mRNAs in the ceRNA regulatory network

Based on the functional enrichment analysis, the 146 mRNAs involved in the identified ceRNA regulatory network were remarkably enriched for 55 GO BP terms and 13 KEGG pathways (FDR < 0.05). The top 10 enriched GO BP terms, mainly including "regulation of phospholipase activity", "extracellular structure organization", "positive regulation of phospholipase activity" and several system development-related terms, are shown in Figure 4C. The top 10 enriched KEGG pathways, mainly including the "Hippo signaling pathway", "AGE-RAGE signaling pathway in diabetic complications", "PI3K-Akt signaling pathway" and several other cancer-related pathways, are shown in Figure 4D.

With the threshold of a minimum required interaction score > 0.4, the STRING database was used to construct a PPI network based on the 146 mRNAs involved in the identified ceRNA regulatory network. After wiping out the isolated nodes in the network, 96 nodes and 180 edges were mapped in the PPI network (Figure 4E). The top 3 mRNAs with the highest degrees were FGF2 (degree = 17), KIT (degree = 13) and NCAM1 (degree = 11).

### Prognosis values of lncRNAs and mRNAs in the ceRNA network

**Figure 5:**
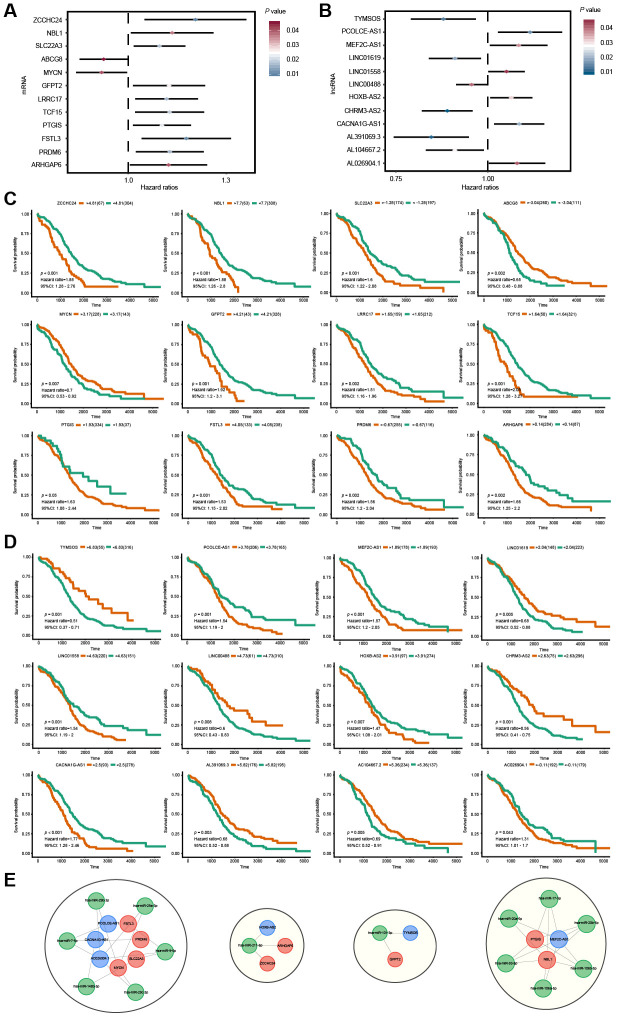
**Survival analysis for lncRNAs and mRNAs in the ceRNA network.** (**A, B**) Forest plots of hazard ratios (HR) of survival-associated mRNAs and lncRNAs in the ceRNA regulatory network. (**C, D**) Kaplan-Meier survival curves for mRNAs and lncRNAs. The horizontal axis indicates the overall survival time in days, and the vertical axis shows the survival rate. (**E**) The OS-specific ceRNA sub-network. The lncRNAs, miRNAs and mRNAs are indicated as blue, green and red, respectively.

To identify the mRNAs and lncRNAs with potential prognostic value in predicting OS, survival analyses of 146 mRNAs and 201 lncRNAs in the ceRNA regulatory network were conducted using the univariate Cox proportional hazards regression model. The results showed that 24 RNAs, including 12 mRNAs and 12 lncRNAs, were significantly associated with OS (Figure 5A, 5B). Among these significant RNAs, six lncRNAs (*TYMSOS*, *LINC01619*, *LINC00488*, *CHRM3-AS2*, *AL391069.3* and *AC104667.2*) and two mRNAs (*ABCG8* and *MYCN*) were negatively associated with OS (*P* < 0.05). In comparison, the remaining six lncRNAs (*PCOLCE-AS1*, *MEF2C-AS1*, *LINC01558*, *HOXB-AS2*, *CACNA1G-AS1* and *AC026904.1*) and ten mRNAs (*ZCCHC24*, *NBL1*, *SLC22A3*, *GFPT2*, *LRRC17*, *TCF15*, *PTGIS*, *FSTL3*, *PRDM6* and *ARHGAP6*) were positively associated with OS (*P* < 0.05). Based on their respective optimal cut-offs, Kaplan-Meier curves of these 12 mRNAs and 12 lncRNAs associated with OS were drawn (Figure 5C, 5D). The results of the Kaplan-Meier curve analysis and log-rank test of these 24 RNAs were proven to be consistent with the results of the univariate Cox regression analysis. Furthermore, we extracted the OS-specific ceRNA sub-network of these 24 RNAs. As shown in Figure 5E, The OS-specific ceRNA sub-network contained 4 RNA molecules, including 6 OS-related lncRNAs, 14 interacting miRNAs and 9 OS-related mRNAs.

### Construction of the OVC-specific lncRNA-based prognostic signature

**Figure 6:**
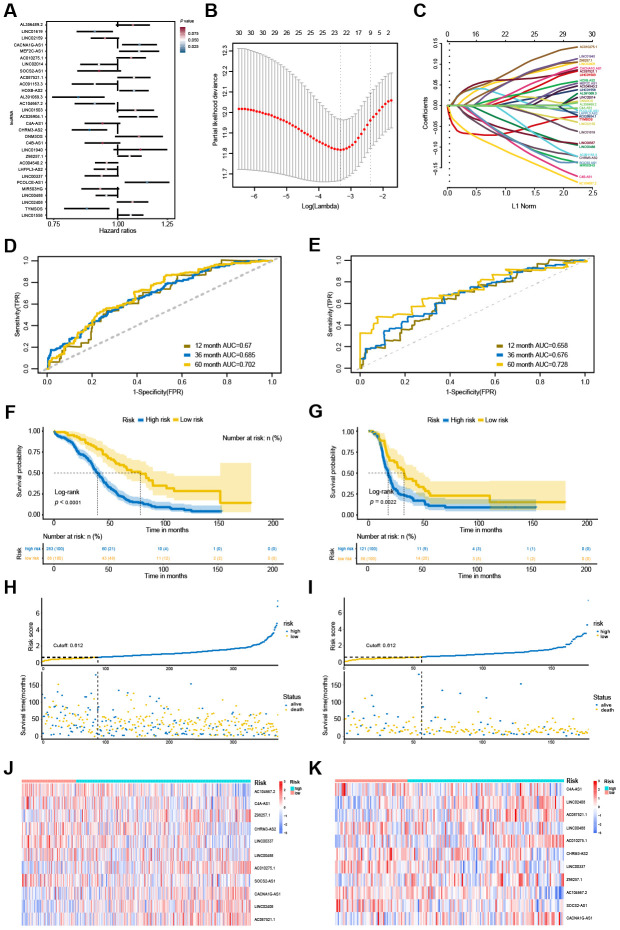
**Identification and performance evaluation of the 11-lncRNA signature.** (**A**) Forest plots of hazard ratios (HR) of the 30 potential OS-associated lncRNAs.(**B**) Selection of the tuning parameter (lambda) by ten-fold cross-validation based on the minimum criteria for OS. (**C**) The coefficient profiles of the 30 potential OS-associated lncRNAs at varying levels of penalty. (**D, E**) The time-dependent ROC curves of the 11-lncRNA signature in predicting OS and DFS. (**F, G**) Kaplan-Meier curves of patients with low or high risk in the TCGA-OS cohort and TCGA-DFS cohort. (**H, I**) Risk score distribution and survival status of patients in the TCGA-OS cohort and TCGA-DFS cohort. (**J, K**) RNA expression heat map of the 11 prognostic signature between low-risk and high-risk groups in the TCGA-OS cohort and TCGA-DFS cohort.

Through the univariate Cox regression analysis, we identified 30 potential OS-associated lncRNAs (Figure 6A). Then, we used the LASSO regression model to further identify an optimal subset of the lncRNA-based signature reliably associated with OS. As a result, 11 lncRNAs, including *C4A-AS1*, *LINC02408*, *AC087521.1*, *LINC00488*, *AC010275.1*, *CHRM3-AS2*, *LINC00337*, *Z98257.1*, *AC104667.2*, *SOCS2-AS1* and *CACNA1G-AS1*, were identified for modeling (Figure 6B, 6C). Furthermore, a lncRNA-based risk score was developed by integrating the expression data for these 11 lncRNAs with corresponding coefficients weighted by the LASSO model as follows: Risk score = (0.147 × *LINC02408* expression value) + (-0.066 × *LINC00488* expression value) + (-0.161 × *LINC00337* expression value) + (0.104 × *Z98257.1* expression value) + (-0.129 × *CHRM3-AS2* expression value) + (-0.14 × *C4A-AS1* expression value) + (-0.156 × *AC104667.2* expression value) + (0.088 × *AC087521.1* expression value) + (-0.159 × *SOCS2-AS1* expression value) + (0.096 × *AC010275.1* expression value) + (0.111 × *CACNA1G-AS1* expression value).

The risk score was calculated for all 371 OVC patients with the lncRNA-based prognostic signature. The accuracy of this prognostic signature in OVC long-term OS predictions was evaluated by ROC curves. The values of the area under the ROC (AUC) for the 1-year, 3-year and 5-year OS prediction models was 0.67, 0.688 and 0.702, respectively (Figure 6D). After estimating the maximally selected rank statistics, patients were divided into two groups based on the optimal risk score cut-off (0.612). Patients with risk scores higher than 0.612 were classified into the high-risk group (283 patients), whereas those with risk scores less than or equal to the cut-off value were allocated to the low-risk group (88 patients). Survival analysis suggested that high-risk patients were at a markedly increased risk of death in OVC and predicted a shorter OS (Figure 6F and 6H). The heatmap revealed that the expression of these 11 lncRNAs varied by risk score (Figure 6J). This prognostic signature also showed an excellent performance to predict disease-free survival (DFS) (Figure 6E, 6G, 6I, 6K). In the TCGA-DFS dataset (n = 177), the AUC for this prognostic signature for the 1-year, 3-year and 5-year DFS prediction models were 0.658, 0.676 and 0.728, respectively (Figure 6E). Based on the same cut-off (0.612), patients in the low-risk group had better survival time than those in the high-risk group and a decreased risk of death (Figure 6G, 6I).

**Figure 7:**
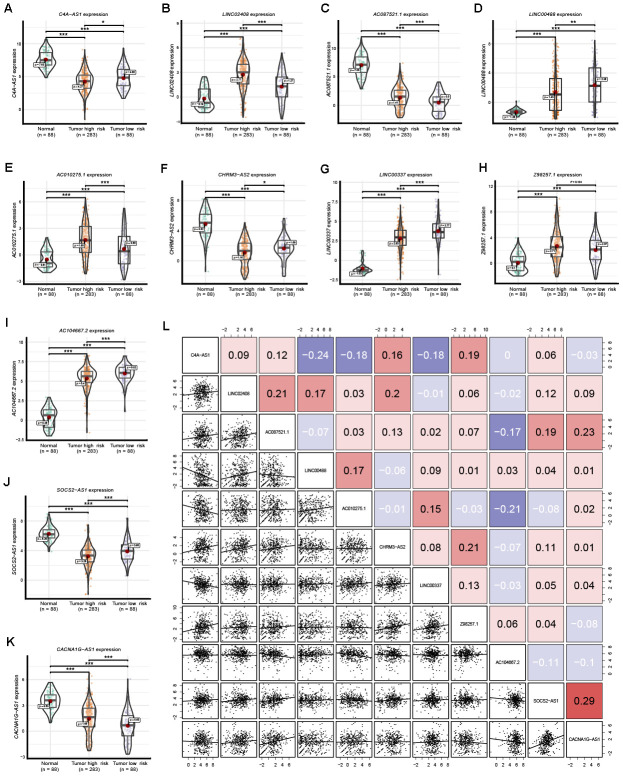
**Expression of the 11 prognostic signature lncRNAs among normal ovarian tissues, SOC tumor high-risk and SOC tumor low-risk groups.** (**A–K**) The expression level of the 11 prognostic signature RNAs among normal tissue, high-risk and low-risk groups. (**L**) Correlation among these 11 prognostic signature RNAs. Numbers indicate the Pearson coefficients.

Expression of the 11 lncRNAs was analyzed among normal tissues, tumor high-risk and tumor low-risk groups. As shown in Figure 7, all 11 lncRNAs among the three groups were significantly differentially expressed. Furthermore, the expression of *Z98257.1* in the high-risk group was not significantly higher than that in the low-risk group; the other ten lncRNAs were significantly differentially expressed between every two-group comparison (Figure 7H). Coexpression analysis revealed that these 11 lncRNAs showed a weak or moderate-intensity of coexpression interactions as evaluated by the Pearson correlation coefficient (Figure 7L).

To investigate whether the prognostic value of the 11-lncRNA signature was independent of other clinical variables, we performed univariate and multivariate Cox regression analysis, which included risk score, age, race, TNM classification, venous invasion and lymphatic invasion as covariates. The results showed that either in the TCGA-OS dataset (HR = 1.36, *P* < 0.0001) or the TCGA-DFS dataset (HR = 1.32, *P* < 0.0001), the 11-lncRNA risk score was an independent prognostic factor (Table 1). In addition, age (HR = 1.02, *P* = 0.01) and race (HR = 0.55, *P* = 0.0052) were also independent prognostic factors of OS (Table 1). Therefore, the 11-lncRNA signature may offer an approach for risk assessment and predict the prognosis of OVC patients.

**Table 1:** Univariate and multivariate Cox regression analysis of the 11-lncRNA signature and clinical factors associated with OS.

	**Univariate analysis**		**Multivariate analysis**	
	**HR (95% CI)**	***P* value**	**HR (95% CI)**	***P* value**
OS cohort (n=371)				
Age	1.02 (1.01, 1.03)	0.00182	1.02 (1.00, 1.03)	0.01
Race (white vs. not white)	0.58 (0.38, 0.88)	0.0114	0.55 (0.36, 0.84)	0.0052
TNM classification (III and IV vs. I and II)	1.99 (0.88, 4.49)	0.0959		
Venous invasion	0.90 (0.49, 1.68)	0.7521		
Lymphatic invasion	1.41 (0.83, 2.38)	0.2055		
Risk score	1.38 (1.25, 1.51)	<0.0001	1.36 (1.23, 1.49)	<0.0001
DFS cohort (n = 177)				
Age	1.00 (0.98, 1.02)	0.9788		
Race (white vs. not white)	0.83 (0.45, 1.56)	0.5696		
TNM classification (III and IV vs. I and II)	1.54 (0.78, 3.03)	0.2144		
Venous invasion	1.09 (0.53, 2.26)	0.8173		
Lymphatic invasion	1.17 (0.62, 2.21)	0.6348		
Risk score	1.32 (1.15, 1.51)	<0.0001	1.32 (1.15, 1.51)	<0.0001

**Figure 8:**
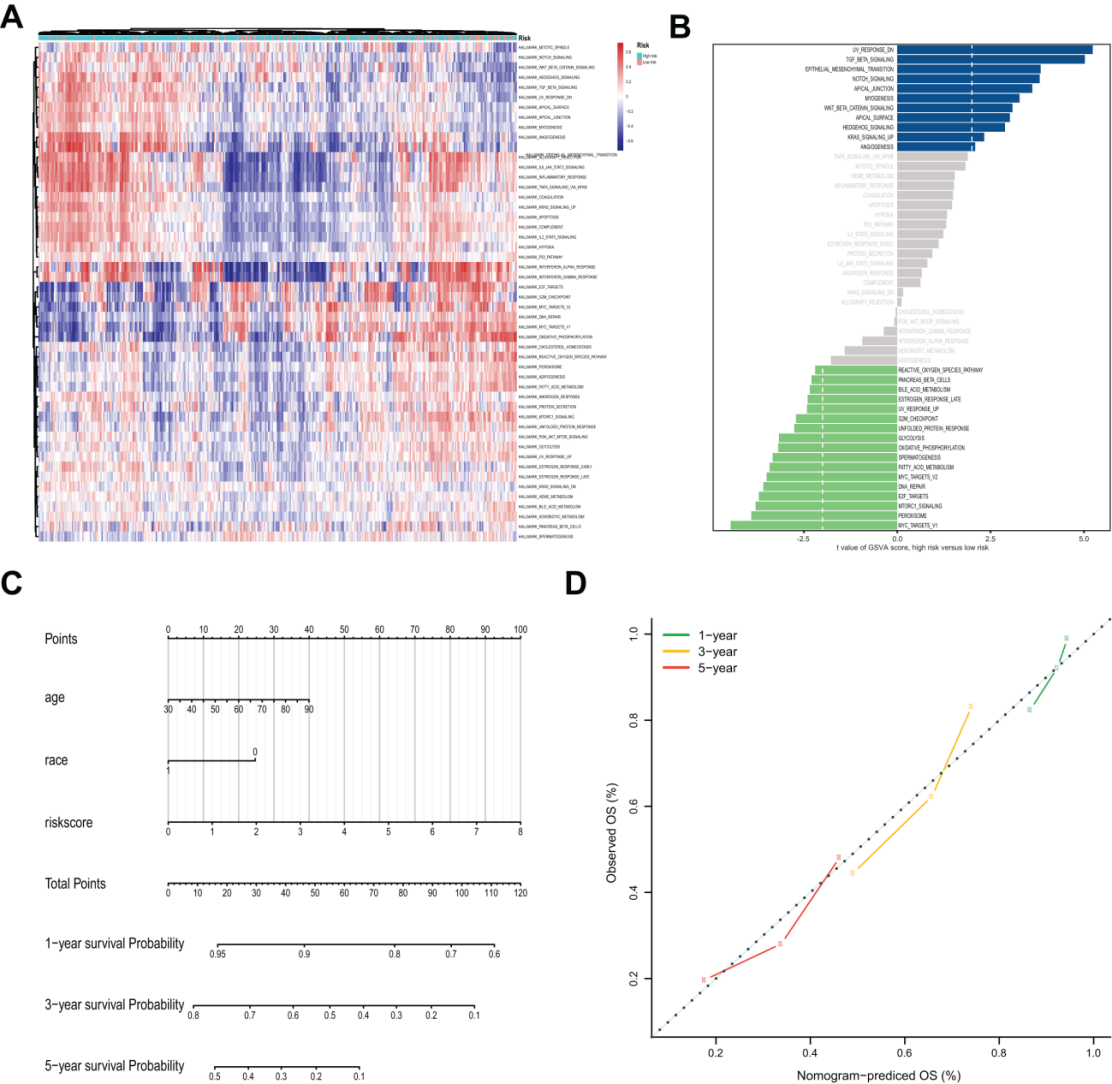
**Gene Set Variation Analysis (GSVA) analysis and nomogram for the prediction of prognosis.** (**A**) The heatmap of GSVA scores of the 50 key pathways between high-risk and low-risk groups. (**B**) Differences in pathway activities scored by GSVA between high-risk and low-risk groups. The blue column indicates activated pathways in the high-risk group, and the green column indicates activated pathways in the low-risk group. (**C**) Nomogram prediction of 1-year, 3-year and 5-year OS. For race, 0 means white, and 1 means not white. (**D**) Calibration curves of observed and predicted probabilities for the nomogram.

The analysis of the hallmark pathway gene sets highlighted that the 50 key signaling pathways in OVC samples were quite different between the high- and low-risk groups (Figure 8A, 8B). Of note, OVC samples in the high-risk group were most significantly enriched for TGF-, NOTCH-, WNT-, HEDGEHOG-, KRAS- and EMT (epithelial-mesenchymal transition) signaling pathways. In comparison, OVC samples in the low-risk group were most significantly enriched for MYC-, MTORC1- and some hormonal fluctuations and metabolism pathways.

### Construction of a nomogram integrating the 11-lncRNA signature and clinical factors

Considering the clinical relevance and prognostic value of age and race, a nomogram based on the 11-lncRNA signature, age and race was established (Figure 8C and Table 1). Calibration curves for estimating 1-year, 3-year and 5-year survival showed that there were good correlations between the prediction made by the nomograms and the actual observation (Figure 8D). The results suggested that the nomogram could accurately predict 1-year, 3-year and 5-year survival of OVC patients.

## DISCUSSION

OVC is the most serious gynecologic cancer and one of the leading causes of female cancer death [24]. Despite rapid developments in surgical technique, radiotherapy and chemotherapy, the overall five-year survival rate of OVC remains poor [25]. Because of the variation in hyphology, OVC is regarded as a highly heterogeneous disease, and this heterogeneity is an obstacle for proper assessment and treatment [24]. Despite this known predicament, standard treatment of surgical resection followed by platinum- and taxane-based chemotherapy are still needed [26]. Frustratingly, recurrence after the initial surgery and first line chemotherapy may result in platinum-resistant diseases, leading to a poor prognosis [27]. Drug-resistant OVC recurrence is conceivably due to the ability of drug-resistant cells to escape from the surgery and first line chemotherapy [28]. Meanwhile, the role of the immune system and surrounding microenvironment in drug-resistant OVC recurrence is nonnegligible. Immunotherapy after “canonical” chemotherapy seems to be a future option [28]. On the other hand, the danger of benign diseases is often overlooked. For example, patients suffering from endometriosis bear a stronger risk of hematopoietic and ovarian cancer [29].

The important roles of the genome and transcriptome in OVC cells have received increasing attention. Substantial evidence has suggested that the disruption of ceRNA interactions plays an essential role in the pathogenesis of OVC. For example, the lncRNA *PTAR* induces epithelial-mesenchymal transition by competitively binding *miR-101-3p* to regulate *ZEB1* expression [23]. The lncRNA *SNHG6* promotes cell proliferation and migration through sponging *miR-4465* to modulate *EZH2* expression [18]. However, previous studies have mainly focused on a single lncRNA-miRNA-mRNA axis with a limited sample size. Understanding the landscape of the OVC-specific ceRNA regulatory network could lead to deep insight into molecular networks and provide potential therapeutic targets. Here, we comprehensively explored the interactions among lncRNA, miRNA and mRNA to elucidate the landscape of the ceRNA network in OVC.

In the present study, we systematically analyzed OVC-related RNA-Seq data from the TCGA and GTEx databases. Overall, 3,279 DElncRNAs and 4,789 DEmRNAs were identified between the OVC tissues and normal tissues by differential expression analysis. Meanwhile, we found that 2,950 lncRNAs and 2,559 mRNAs were most significantly associated with OVC tumor status by WGCNA. To improve reliability, the OVC-specific RNAs were defined as the intersecting RNAs of these two approaches. Finally, 1,885 lncRNAs and 1,827 mRNAs were determined to be OVC-specific RNAs, which were further used to build the ceRNA regulatory network.

According to the ceRNA theory, we constructed a lncRNA-miRNA-mRNA regulatory network, including 201 lncRNAs, 85 miRNAs and 146 mRNAs. Then, we performed functional enrichment analysis and built the PPI network to explore the potential mechanism of the ceRNA network. As expected, the target genes were mainly enriched for pathways related to OVC. For instance, the "PI3K-Akt signaling pathway" was the most frequently altered intracellular pathway in OVC [30, 31]. "Hippo signaling pathway", "MAPK signaling pathway" and "Ras signaling pathway" were also associated with the progression of OVC, which further supports the accuracy and reliability of the results of our enrichment analysis [32–34]. Of note, we found that the target genes in the ceRNA regulatory network were also enriched for pathways associated with many other cancers, such as breast cancer, melanoma, gastric cancer, and small cell lung cancer. This indicated that the ceRNA regulatory network in our study was universal in human cancer and might play an essential role in the tumorigenesis and development of multiple cancers. From the PPI network, we found that most mRNAs had protein interactions with each other, and the top 3 hub genes in the PPI network were FGF2, KIT and NCAM1, all of which have been reported as OVC-associated genes [35–37]. Finally, the relationships between the expression of these RNAs and OS were investigated. As a result, 12 mRNAs and 12 lncRNAs in the ceRNA regulatory network were identified to associate with OS in OVC patients. Interestingly, these 24 OS-related RNAs formed 4 OS-specific ceRNA molecules. We believe that in-depth insight into the regulatory mechanisms of the 4 OS-specific ceRNA molecules could inform new approaches to the treatment of OVC.

Subsequently, we identified an 11-lncRNA-based signature using LASSO regression, which showed excellent performance in the prediction of both the OS and DFS of OVC patients. Multivariate Cox regression analysis determined that this 11-lncRNA-based signature was an independent prognostic indicator for both OS and DFS. The ROC curves showed that this prognostic signature could effectively predict both early and late survival, all of which suggested that our prognostic signature was closely associated with the prognosis of OVC.

Among the 11 lncRNAs, their expression was significantly different among normal tissues, tumor high-risk and tumor low-risk groups or even between every two groups. This indicated that all 11 lncRNAs might serve as critical regulatory factors in the tumorigenesis and development of OVC and could therefore act as novel biomarkers and therapeutic target candidates. Consistent with our results, lung adenocarcinoma patients with highly expressed *AC087521.1* had a shorter survival time [38]. Highly expressed *LINC00488* was significantly associated with prolonged survival for patients with colorectal cancer [39, 40]. *SOCS2-AS1* was highly expressed and correlated with a high survival rate in colorectal cancer patients [41]. The overexpression of *CACNA1G-AS1* in hepatocellular carcinoma patients was linked to a low survival rate [42]. Of note, *SOCS2-AS1* and *CACNA1G-AS1* can act as a ceRNA to participate in tumorigenesis and development [41, 42]. These studies verified the accuracy of our results, which increased the credibility of our study.

Finally, a prognostic nomogram was developed based on independent prognostic factors associated with OS. This nomogram could accurately predict the 1-year, 3-year and 5-year survival of OVC patients. A low score represents a prolonged OS, while a high score indicates a poor OS. In clinical practice, if the patients have a higher score, closer follow-up and adequate treatment are necessary. Our GSVA analysis also revealed the different molecular mechanisms between low-risk and high-risk groups, which provided the basis for individualized treatment plans.

The novel OVC risk formula will be beneficial to the tailored individualized therapy and prognosis of OVC patients. Along with the development of a prognostic surveillance system, the changing state of the disease could be monitored by the doctor. At the same time, synthetic anticancer drugs are increasingly being developed and prepared for clinical application, such as solasodine acetate and N7mdG [43, 44]. To prevent cancer, the concept of a cancer vaccine was proposed. Nanoparticles and nanomaterials have been promised as delivery vectors for cancer vaccines [45].

In conclusion, our study depicts a landscape of the lncRNA-miRNA-mRNA ceRNA from multiple dimensions and provides serval candidate biomarkers and therapeutic targets for OVC patients. Moreover, an 11-lncRNA-based signature was constructed to predict the prognosis of OVC, which might serve as a therapeutic decision marker. Our findings provide novel insight into the lncRNA-related regulatory mechanism of the ceRNA network in OVC and might be the foundation for future basic and clinical research.

## MATERIALS AND METHODS

### Patients and samples

The RNA-Seq data and clinical phenotype information of OVC tissues obtained from the TCGA project and normal tissues obtained from the GTEx project were downloaded from the UCSC Xena database (http://www.genome.ucsc.edu) [46]. All the OVC patients were selected according to the following criteria: (1) patients had a histopathological diagnosis of OVC; (2) patients had no other tumors; (3) patients did not receive preoperative chemoradiation; (4) patients had complete RNA-Seq data and clinical prognosis data. Finally, 459 samples, including 371 OVC tissues and 88 normal tissues, were enrolled in our study. This study was performed following the TCGA and GTEx publication guidelines. As the data were all retrieved from TCGA and GTEx, approval from a local Ethics Committee was not necessary.

### Data preprocessing

The mRNAs and lncRNAs were identified and annotated from the RNA-Seq data via the Ensembl database (http://www.ensembl.org), and miRNAs were annotated based on the miRbase database (http://www.mirbase.org) [47, 48]. The RNA-Seq data that could not be annotated in the database were discarded. The expression of lncRNAs, mRNAs and miRNAs with zero counts in more than 20% of samples was filtered out to remove low-abundance genes. [49, 50].

### Differential expression analysis

Differentially expressed lncRNAs (DElncRNAs) and mRNAs (DEmRNAs) between OVC tissues and normal tissues were analyzed using the "edgeR" R package [51, 52]. *P-*values were corrected with a false discovery rate (FDR). Fold changes of expression levels (log2 absolute) ≥ 2 and FDR < 0.05 were set as the thresholds.

### WGCNA

To identify the significant lncRNAs and mRNAs associated with the carcinogenesis of OVC, WGCNA analysis was performed according to the protocol and recommendations of the "WGCNA" R package [53] to construct independent signed networks and establish the weighted gene coexpression modules and module-trait relationship from the SOC samples and normal samples. The scale-free topology fitting index (R^2^) > 0.85 was set as the threshold to construct the weighted gene coexpression network. A minimum cluster size of 30 and a merge threshold function of 0.25 were chosen as the thresholds to identify coexpressed gene modules. A biweight mid-correlation coefficient (r) ≥ 0.8 and P-value < 0.05 were set as the thresholds to determine statistically significant modules with the carcinogenesis of OVC.

### Functional enrichment analysis of the overlapped mRNAs

Enrichment analysis was performed to understand the potential mechanism and function of the overlapped mRNAs. Gene Ontology (GO) terms, including molecular function (MF), biological process (BP) and cellular component (CC) categories, and Kyoto Encyclopedia of Genes and Genomes (KEGG) enrichment analyses were performed using the "ClusterProfiler" R package, with FDR < 0.01 as the threshold [54].

### Construction of the lncRNA-miRNA-mRNA regulatory network

Based on the ceRNA hypothesis, the lncRNA-miRNA-mRNA ceRNA network was constructed for the overlapped mRNAs and lncRNAs through "GDCRNATools" following the following three steps: (1) miRcode (http://www.mircode.org/) database was used to predict the lncRNA-miRNA interactions, and miRTarBase database (http://mirtarbase.mbc.nctu.edu.tw) was used to predict the miRNA-mRNA interactions; (2) according to the common miRNA, the lncRNA-miRNA and miRNA-mRNA interactions were merged into the potential lncRNA-miRNA-mRNA ceRNA triples. Moreover, the hypergeometric test was used (*P*-value < 0.05 for the hypergeometric tests) to determine the significance of the shared miRNAs by lncRNA and mRNA pairs; (3) according to the ceRNA hypothesis, in the lncRNA-miRNA-mRNA ceRNA triples, the expression of lncRNA and mRNA pairs should be positively correlated, and the expression of lncRNA-miRNA pairs and miRNA-mRNA pairs should be negatively correlated. The Pearson correlation analysis was performed (*P*-value < 0.05 for the Pearson's correlation tests) to measure the expression correlation of lncRNA-miRNA, miRNA-mRNA and lncRNA-mRNA pairs [55–57]. Cytoscape (version 3.7.1) software was used to construct and visualize the lncRNA-miRNA-mRNA regulatory network [58].

### Functional enrichment and protein-protein interaction (PPI) analyses of the mRNAs in the ceRNA network

To understand the underlying pathways and processes of the ceRNA regulatory network, the "ClusterProfiler" R package was used to analyze the functional profiles (GO BP terms and KEGG pathway) of the mRNAs in the ceRNA regulatory network [54]. With a threshold of FDR < 0.05, the results of the GO BP and KEGG enrichment analyses were displayed using the "GO plot" R package. To identify the protein-protein interactions between the mRNAs in the ceRNA regulatory network, STRING (Search Tool for the Retrieval of Interacting Genes) database (http://www.string-db.org/) was used to construct a PPI network (minimum required interaction score > 0.4), which was visualized by Cytoscape (version 3.7.1) software [58, 59]. By ranking the degree of connectivity between the mRNAs in the PPI networks, high-degree genes, the so-called "hub" genes, were identified using the Cytohubba plugin [60].

### Survival analysis of lncRNAs and mRNAs in the ceRNA regulatory network

Univariate Cox proportional hazards regression analysis was performed to estimate the expression of lncRNAs and mRNAs in the ceRNA regulatory network with overall survival (OS) through the "survival" package in R software. Based on the gene expression of each patient with optimal cut-off values, which was determined by the "survminer" R package, the patients were divided into high-expression and low-expression groups. Then, using the "survival" R package, Kaplan-Meier curves and log-rank methods (Mantel-Haenszel test) were used to examine the statistically significant OS-related mRNAs and lncRNAs, which were determined by the univariate Cox regression analysis. *P*-value < 0.05 was considered statistically significant.

### Construction of lncRNA-associated prognostic signatures

The potential OS-related lncRNAs chosen from the univariate Cox regression analysis (*P*-value < 0.1) were inserted into the least absolute shrinkage and selection operator (LASSO) analysis to calculate the coefficients. Ten-fold cross-validation was conducted to tune the optimal value of the penalty parameter λ, which gives the minimum partial likelihood deviance. According to the linear combination of the expression values weighted by the regression coefficient from the LASSO analysis, risk scores for each sample were calculated. All patients were classified into either the high-risk or low-risk group based on the optimal cut-off value of their risk score. The optimal cut-off value was identified using the "survminer" R package. The survival curve in the high-risk and low-risk groups was estimated and compared by the Kaplan-Meier method and the log-rank test. A *P-*value < 0.05 was considered statistically significant. The receiver operating characteristic (ROC) curves and areas under the ROC curves (AUC values) were performed to evaluate the sensitivity and specificity of the risk score model for survival prediction using the "survival ROC" R package.

Multivariate Cox regression analysis was performed to determine whether the risk score model was independent of other clinical features, including age, race, TNM classification, venous invasion and lymphatic invasion. The hazard ratio and 95% confidence intervals for each variable were calculated. A *P*-value < 0.05 was considered statistically significant.

### Gene Set Variation Analysis (GSVA)

To further explore the biological function of the 11-lncRNA signature in the occurrence and development of OVC, we performed GSVA between high-risk and low-risk groups using the "GSVA" R package [61]. Each gene set associated with a pathway was trimmed to only contain unique genes to reduce pathway overlaps and pathway redundancies [62]. The hallmark gene sets were used as the reference gene set [63]. FDR < 0.05 was set as the cut-off criterion.

### Construction of nomogram predictive models

To precisely predict the 1-year, 3-year and 5-year OS of OVC patients, a prognostic nomogram was established using the "rms" R package based on the 11-lncRNA signature, age and race. Calibration curves were used to compare the concordance between nomogram-predicted survival and observed survival.

### Statistical method

For the correlation analysis, Pearson's correlation test was performed. For the survival analysis, Cox regression analysis and Kaplan-Meier log-rank test were performed. To analyze differences between groups, the Mann-Whitney test was used for continuous data, whereas the Chi-square test was used for categorical data.
